# Connection between nutrition and oncology in dogs and cats: perspectives, evidence, and implications—a comprehensive review

**DOI:** 10.3389/fvets.2024.1490290

**Published:** 2025-02-18

**Authors:** Andressa R. Amaral, Gabriela L. F. Finardi, Pedro H. Marchi, Natália M. C. de Oliveira, Leonardo A. Príncipe, Natacha Teixeira, Maria C. F. Pappalardo, Laís O. C. Lima, Juliana V. Cirillo, Júlio Cesar de C. Balieiro, Thiago H. A. Vendramini

**Affiliations:** 1 Veterinary Nutrology Service, Veterinary Teaching Hospital of the School of Veterinary Medicine and Animal Science, University of São Paulo, São Paulo, Brazil; 2 Pet Nutrology Research Center, Department of Animal Nutrition and Production of the School of Veterinary Medicine and Animal Science, University of São Paulo, Pirassununga, Brazil

**Keywords:** appetite, cachexia, cancer, canine, feline, food, nutrology

## Abstract

Oncology has become one of the most influential and studied areas in both human and companion animal health. In veterinary practice, cancer represents a significant challenge, especially concerning cats and dogs. Nutrition plays a crucial role in the management of oncology patients in veterinary medicine; however, is often nonspecific and reliant on data from other species and diseases, highlighting the need for a comprehensive review of the latest developments in this field. Since the intricate relationship between nutrition and cancer encompasses various aspects, this review therefore intends to cover the most important points in nutrition in canine and feline oncology. Therefore, topics are addressed that include discussion about the effects of cancer on nutrition, cancer-related cachexia, the influence of obesity on both the occurrence and progression of cancer, essential nutrients for oncologic patients, and nutritional supplementation.

## 1 Introduction

Cachexia is characterized primarily by loss of muscle mass and is a common and serious syndrome in cancer patients (1). It results not only from reduced caloric intake, but from complex metabolic changes induced by cancer, which lead to the degradation of muscle and adipose tissue (2). Early identification of this syndrome is challenging, but essential for effective management. Carefully formulated diets that limit the energy available to cancer while maintaining the patient’s needs and promoting anti-inflammatory and immunomodulatory effects, can improve the quality of life and prolong the survival of affected animals (3). Nonetheless, the management of cancer cachexia in companion animals is complex, especially due to loss of appetite, one of the main challenges that directly affects the quality of life of animals and worries their owners (4). This lack of appetite is multifactorial and can be caused both by the disease itself and by the side effects of medications. Cytokines such as IL-1, IL-6 and TNF-*α* play central roles in orexia regulation, often promoting anorexia by activating mechanisms that inhibit appetite (5). At the same time, studies indicate that obesity in animals, as in humans, is associated with a chronic inflammatory state that can facilitate the development of neoplasia (6).

Early identification of cachexia-sarcopenia-malnutrition syndrome is essential to understand protein intake and amino acid supplementation in cancer patients, aiming to slow the loss of lean body mass (7). Adequate intake of proteins and specific amino acids, such as leucine and lysine, aids in maintaining lean mass and improving the immune response, which is particularly important for dogs and cats with cancer (8). Studies indicate that increasing protein intake can prevent muscle degradation, and amino acid supplementation can optimize the nutritional and immunological status of these patients (9, 10).

Cancer patients may require greater energy support due to the hypermetabolism associated with the disease; thus, carbohydrates serve as an important source of calories (11). Although previously considered harmful to cats and dogs, these species have evolved to the point where they are effective at metabolizing carbohydrates, with no evidence that these nutrients can cause metabolic syndrome (12, 13). In cats and dogs with cancer, an adequate carbohydrate intake may mitigate paraneoplastic cachexia, despite increased glucose uptake by cancer cells (14). Ketogenic diets and carbohydrate reduction have been studied, but the total absence of these nutrients may be harmful (15). It is recommended to balance carbohydrate intake according to their glycemic index, to benefit the patient without stimulating tumor growth. Although there are no direct studies linking fiber intake to cancer in dogs and cats, the inclusion of fiber in the diet of these animals is recommended for intestinal health and can be explored in future research to optimize treatment (16).

In contrast, fatty acids are essential nutrients for dogs and cats, playing crucial roles in several metabolic functions, including inflammatory response (17). The high caloric density of these nutrients makes them ideal for meeting energy needs with a smaller food volume, which is especially useful for cancer patients in a hypermetabolism state. In addition, supplementation with omega-3 fatty acids has shown positive effects, such as modulation of inflammation and potential reduction in tumor growth and metastasis (18), although further studies are needed to confirm these benefits in animals.

Regarding vitamins and minerals, specific clinical studies on supplementation in dogs and cats with cancer are still scarce, and there is no recommended dose for these cases. In humans, for example, vitamin D deficiency is associated with the development and progression of several types of cancer (19), and supplementation can reduce cancer mortality (20). Such as high doses of vitamin C have shown anticancer effects, but its efficacy remains controversial (21). Although vitamin C is essential for humans, it is not essential for healthy cats and dogs, as they synthesize it in the liver (22).

Furthermore, zinc is involved in several biological processes and its deficiency is associated with cancer in humans and animals (23). Studies indicate that low zinc levels may be linked to certain types of cancer in dogs (24), such as lymphoma and osteosarcoma. Selenium and vitamin E have also been investigated for their potential anticancer effects (25, 26), but the results are conflicting, and more research is needed to confirm their efficacy and safety.

Thus, this review aims precisely, through a comprehensive review and always based on science and research, to bring an initial and necessary discussion about possible nutritional interventions in canine and feline oncology.

## 2 The cancer patient

### 2.1 Cancer effects in nutrition

Among the many difficulties in the nutritional management of cancer patients, alleviating the effects of cancer on appetite is one of the main focuses to bring any remaining possibility of cure or quality of life (27). This is an even greater concern in veterinary medicine, where strategies to overcome it do not include conversational ability; thus, hunger or even the willingness to eat must be present.

Loss of appetite is also one of the main causes of pet owners’ concern, since this is one of the few variables that he is able to interfere within the day-to-day care, and it is associated with their interpretation of health and well-being (28). Studies with primary veterinary care have pointed out that 14% of dogs referred for consultation are due to inappetence symptoms (4). Research evaluating the factors that influence owners on pursuing chemotherapy found that vomiting, increased sleepiness and reduced activity and playing were considered acceptable. However, inappetence, weight loss and depression were not. Thus, among most side effects, not eating is intolerable and influences end-of-life decisions (28).

Inappetence in dogs and cats with cancer is multifactorial and may be primarily related to the disease or drug treatment and its adverse effects (29). However, since side effects of medications are not a field for nutritional intervention, the understanding of the underlying causes of loss of appetite due to the disease itself is the main target for nutrition assistance (30).

The overall hunger and satiety signaling rely on the perception that food has reached the gastrointestinal tract and glucose has entered the cells (31). In this context, the physiology of appetite is more influenced by the negative feedback (stop eating when there is food) than mechanisms for increase energy intake (32). For this reason, some authors consider only ghrelin as the main appetite stimulant (33) and stretch receptors in the gut, such as cholecystokinin, peptide Y, insulin, and leptin, are considered responsible for decreasing food intake (34). Thus, there are more mechanisms involved in stopping eating than in stimulating appetite (4).

The causes of anorexia may be directly related to dysphagia, hypoxia and alteration of gastrointestinal function, such as motility and capability to digest, or related to inflammatory process mediated by tumor necrosis factor alpha (TNF-*α*), interleukin 6 (IL-6) and interleukin 1 (IL-1) and 1 beta (IL-1β) (35).

IL-1 is a cytokine primarily known for its role in the immune system and has gained attention due to its effects on appetite regulation (36). Studies with animal models observed that administration of an IL-1β receptor antagonist increased meal number in rats (37). Additionally, IL-1 acts at the central nervous system, particularly in the hypothalamus where appetite regulation is regulated (38). Studies suggest that IL-1, specifically its subtypes IL-1α and IL-1β, exerts suppressive effects on appetite via multiple pathways (36). One mechanism involves the activation of pro-opiomelanocortin (POMC) neurons, which are anorexigenic. This activation leads to the release of *α*-melanocyte-stimulating hormone (α-MSH), acting as a neurotransmitter after binding and activating melanocortin receptors (MC4R) located on neurons in several brain regions, including the paraventricular nucleus (PVN) of the hypothalamus (39). Activation of MC4R inhibits the activity of orexigenic neurons and stimulates the activity of anorexigenic neurons, ultimately leading to a decrease in food intake (37).

Also, IL-1 and IL-6 has been shown to modulate leptin signaling pathways by influencing the expression and secretion of leptin from adipocytes (40). These cytokines also affect the sensitivity of leptin receptors in the hypothalamus, thereby altering the responsiveness of neurons to leptin and inducing the release of cholecystokinin (CCK) (37, 41). IL-6 is produced by T cells and adipocytes, and increases gradually during the early stages of cachexia, rising dramatically before death (41). Its effects on feeding are well documented, however both pharmacological blockade of central IL-1 or IL-6 signaling, and genetic deficiency of IL-1 receptor or IL-6 receptor have resulted in attenuated food intake and weight loss. A simultaneous blockade of both IL-1 and IL-6 further enhanced the attenuation of GLP-1 receptor, which leads to suppression of feeding behavior (38).

TNF-*α* is primarily produced by activated macrophages (42), although it can be secreted by many other cell types, including adipocytes, endothelial cells, and certain immune cells. It plays a central role in the immune response to infection and inflammation, where it regulates the recruitment and activation of immune cells to sites of injury or infection (43). The anorexigenic effects of TNF-*α* occur after binding to its cognate receptors, TNFR1 and TNFR2, leading to the activation of signaling pathways, including NF-κB and MAPK pathways (44). NF-α can modulate the activity of neuronal populations, including those expressing pro-opiomelanocortin (POMC) and neuropeptide Y/agouti-related peptide (NPY/AgRP), which are critical for appetite control (45).

Also, TNF-*α* can influence the secretion of adipose-derived hormones, such as leptin and adiponectin, which play key roles in appetite regulation and energy homeostasis. Moreover, TNF-α can impact the function of other peripheral tissues, including the gastrointestinal tract, where it may modulate gut hormone secretion and nutrient absorption, consequently influencing appetite (8, 46).

Altogether, evidence showed that these cytokines are also involved in alterations of sensory function in the *chorda tympani* (involved in the transduction of taste) (47, 48). Human patients with cancer have greater sensitivity to bitterness that correlates positively with IL-1β, IL-6 and TNF-*α* concentrations (49, 50). People with advanced stages of cancer and treated with chemotherapy mention decreased sense of smell of the food (46). Besides these cytokines, some emerging signaling mediators are gaining some focus in the anorexia-cachexia syndrome of cancer. In a review by Talbert et al. (51), the authors bring attention toward growth differentiation factor-15 (GDF-15), activin A and lipocalin-2.

Activin A and GDF-15 are produced by some type of tumors and are believed to regulate feeding in humans and were described in cases of pancreatic and other solid tumors. Receptors of GDF-15 are present in the appetite regulation site and when antibodies anti-GDF-15 were effective in controlling nausea and anorexia in some phase 1 clinical trials (51). Regarding activin A, Kubota et al. (52) found that injection of this cytokine in the third ventricle of male rats decreased food and water intake; however, the mechanisms involved in this effect is still to be understood. Activin A is increased in humans cancer patients with cancer cachexia syndrome and its concentrations in blood are positively correlated to muscle loss (53). As GDF-15, these effects are reduced with the blockade of activin A receptor in animal models (54). Finally, lipocalin-2 is another factor produced by tumors and have been described in human patients with pancreatic cancer. Lipocalin-2 may bound to MCR4, that is an important regulator of appetite, and induces anorexia in mice (55).

### 2.2 Undernutrition and cancer

Amid the most frequent conditions in paraneoplastic syndromes in cancer patients, cachexia stands out, characterized primarily by loss of muscle mass (56). In humans, the prevalence of developing cancer-associated cachexia ranges from 31 to 87% (57–59), and in 20% of cases it is the main cause of death (59). For companion animals, the frequency of muscle loss related to neoplastic diagnoses is substantial (60). However, the low incidence of records, the lack of criteria for a concise diagnosis, and the prevalence of owners opting for euthanasia before the syndrome becomes apparent contribute to the limitation of precise data (4).

Dogs and cats with cancer-associated cachexia present reduced quality of life, response to treatment and a lower rate of disease remission when compared to patients who do not exhibit cachexia (61). Among the commonly reported first signs are anorexia, loss of appetite, fatigue, low immunity, and weight loss, which often appear before the decline in muscle mass becomes clinically apparent (62). The weight reduction due to cachexia does not occur simply due to a negative caloric balance, which normally result in fat utilization as metabolic fuel (63). In dogs and cats affected by cancer, there is an increase in the plasma concentration of inflammatory mediators, including inflammatory catecholamines and cytokines, along with stress-related hormones such as cortisol, insulin, and glucagon, which interfere with the ability to use fat as the main energy source (64). Thus, to meet basal energy needs metabolism resorts to available amino acids, rapidly catabolizing muscle reserves (65). Consequently, the loss of lean mass is a hallmark of cachexia, already identifiable in early clinical stages. In more advanced stages, adipose tissue and bones are consumed, leading to apparent depletion of various body compartments (65).

The metabolic changes caused by cancer also influence glucose utilization (63). Neoplasia consumes glucose to generate energy through anaerobic metabolism, resulting in lactate as the final product and promoting an acidic environment that favors tumor growth. The reconversion of lactate to glucose via the Cori cycle is the animal organism’s response, to prevent acidification of the environment (63). However, this results in negative energy balance to the host and production of reactive oxygen species (ROS) that harms the healthy tissue but not the tumor cells, which have high ability to produce antioxidants through an intermediate metabolite from the lactate formation (NADPH ➔ NADP + H^+^) (66).

According to Michael et al. (67), out of 100 dogs with cancer evaluated in their experiment, 15% showed moderate to severe muscle loss. Also, among 64 dogs whose body weights were available before diagnosis, 37% lost more than 5% of their body weight when re-measured at the time of diagnosis. Meanwhile, Baez et al. (68) analyzed 57 cats with cancer, finding that 91% experienced a reduction in muscle tissue. Another crucial finding in the same study indicated that cats with a body condition score (69) of less than 5 had a median survival prognosis of only 3.3 months when compared to cats with a body condition score greater than or equal to 5 (16.7 months).

Identifying lean mass reduction remains a challenge in veterinary medicine, especially in the early stages of the syndrome, due to the lower detectability of signs (70). Therefore, diagnosing cachexia solely based on weight loss can lead to underdiagnosis and consequently errors in the treatment of the already debilitated patient (66). The use of frequent body condition assessment and more sophisticated techniques, such as dual-energy X-ray absorptiometry (DEXA) and bioimpedance analysis, although still having limitations, allows for better accuracy when diagnosing (71). As it is a multifactorial syndrome, simplistic suggestions regarding preventive treatment methods are uncertain in terms of results (66). However, concerning correlated metabolic manifestations, appropriate nutritional management can be an important ally.

The use of foods that prevent energy supply to the tumor but meet the patient’s needs is considered ideal (72). At one point, diets with low carbohydrate level and high lipid content were thought to be the best strategy, as it hinders the consumption of glucose by cancer cells and provides the necessary energy for metabolic functions by using fat as the main energy source (73). Moreover, high protein content supports the maintenance and reconstruction of lean mass, which is compromised by the tumor’s amino acids catabolism (61). In this context, ketogenic diets could be an option for human beings, however it could lead to undernutrition as well, due to the insufficient intake of some nutrients (74).

Later, other authors raised the hypothesis that the carbohydrate intake was not the main concern, but the glycemic index of these (75–77). A higher glycemic index can stimulate the overload production of insulin that leads to the secretion of anabolic hormones such as insulin-like growth factor type 1 (IGF-1) and growth hormone (GH), whose receptors are overexpressed by many tumors (77). This would not be a problem for dogs and cats, since their nutrient and energy needs do not rely on carbohydrates as in humans. Also, the insulin/IGF-1 pathway and its consequences are not a consensus for all tumors in dogs; thus, it could play a role or not depending on the type of tumor (78–81).

Nutraceutical addition to the diet of oncology patients is also encouraged, and can promote anti-inflammatory and immunomodulatory effects (82, 83). Ogilvie (84) evaluated the effects of adding fish oil and arginine to the diet offered to dogs with lymphoma undergoing doxorubicin chemotherapy treatment. The serum lactate concentrations in the animals remained within normal levels, which not only reduced lactatemia caused by tumor cells but also led to improved remission and extended patient lifespan.

Identifying and treating appetite loss is crucial for the success of nutritional management (4), and encouragement for the patient to eat should be provided by both owners and veterinarians. Implementing adjustments to the diet, nutritional support, or other nutritional approaches generally brings benefits in promoting food intake and improving the quality of life of these animals (66).

### 2.3 Obesity and cancer

The main associations between nutrition and cancer are loss of appetite and malnutrition, for reasons previously mentioned in this review. In fact, throughout the progression of the disease in oncological dogs and cats, the tendency is to observe these clinical manifestations (4). However, obesity is also a condition of utmost importance in oncological nutrition (85). Such condition is characterized by abnormal or excessive accumulation of adipose tissue, that poses significant threats to animal well-being and health (86).

Studies conducted in the 2000s in the United States and Australia indicated that approximately 29 to 33.5% of dogs were overweight, with 5.1 to 7.6% classified as obese (87, 88). Recent research in Brazil and Japan has shown a rise in obesity rates, with estimates ranging from 25.9 to 39.8% for overweight dogs and 14.6 to 15.1% for obese ones (89, 90). While less extensive, findings from the United States in 2005 suggested a prevalence of overweight or obese cats at 35%, increasing to 39% in a 2010 study (88, 91).

In human medicine, obesity leads to higher morbidity and mortality rates, and shorter life expectancies (92). Similarly, obese dogs face reduced life spans and quality of life, along with increased risks of various comorbidities, including orthopedic disorders, cardiovascular disease, respiratory issues, insulin resistance, and certain types of cancer (93, 94). Cats also experience health complications such as hepatic lipidosis, lower urinary tract diseases, dermatological conditions, insulin resistance, diabetes, and heightened anesthesia risks (88). Understanding white adipose tissue’s role as an endocrine organ in obese animals has deepened insights into obesity’s pathophysiology and its links to other diseases (95, 96).

White adipocytes regulate physiological functions like appetite, immune responses, and metabolism (97, 98). However, in obesity, excessive weight gain leads to white adipose tissue dysfunction, resulting in unregulated secretion of adipokines, including pro-inflammatory ones, fostering a chronic low-grade inflammatory state (99). This dysfunctional expansion of adipose tissue further stimulates immune responses, aggravating inflammation through a vicious cycle (100). In the current literature, there are many hypotheses that correlate obesity with the development of cancers, worsening of the disease, an increase in the number of metastases, and a reduction in treatment success. However, the main link between these conditions is chronic adipose tissue inflammation (101). In addition to causing constant cellular injury, chronic inflammation creates a microenvironment very similar to that of cancers, which favors the establishment, infiltration, and growth of neoplasms (102).

In human medicine, obesity is strongly associated with the development of various cancers, including endometrial, esophageal, pancreatic, renal, hepatocellular, meningioma, multiple myeloma, and several others (103). There are not many studies on the relationship between obesity and cancer in dogs. In 1989, Glickman et al. (104) observed that the risk of developing transitional cell carcinoma of the bladder was greater in obese dogs regardless of chemical exposures (105). Later, Weeth et al. (105) identified an association between obesity and mast cell tumors. Over the years, research has focused on the relationship between obesity and mammary gland tumors, as these tumors are the most prevalent form of neoplasia and cancer in female dogs (106).

In obese humans, excess white adipose tissue results in increased levels of estrogen (107). The inflammation caused by obesity can interfere with estrogen signaling, leading to DNA damage, cell proliferation, angiogenesis, and mutagenesis in various types of neoplasms (108). Additionally, white adipose tissue produces an excess of aromatase, which is responsible for converting androgenic hormones into estrogen, further elevating its plasma concentrations. In obese female dogs, hormonal carcinogenesis also appears to be a key mechanism for understanding the relationship between obesity and the development of mammary gland tumors (109–114).

In cats, studies are exceedingly limited, making comprehensive research challenging. A cancer of particular interest in cats is feline mammary cancer. This neoplasia is the third most common in cats, making up 12–40% of all tumors identified in cats (115, 116). This type of cancer shares many commonalities with human breast cancer; both diseases show similar molecular classification, epidemiology, and clinicopathological features (115, 117, 118). As there is a high degree of similarity between feline mammary cancer and breast cancer, and since breast cancer is well known to be influenced by obesity, it is important to determine whether obesity also influences the development of feline mammary cancer (117–119). There are several molecular mediators of obesity that also contribute to cancer development. Some of the proteins and hormones that are of interest due to the existing evidence of their role or potential role in feline mammary cancer include leptin and leptin receptor, adiponectin, estrogen, and prolactin (119).

Malnutrition is a clinical manifestation associated with lower survival rates in dogs and cats with cancers (120). However, being overweight and obese is also a problem in oncologic nutrition, as it promotes the development and worsening of various tumors. Therefore, an important nutritional tool for cancer prevention in companion animals is maintaining a healthy and ideal body condition.

## 3 Fundamental nutrients for cancer patients

### 3.1 Carbohydrates

Cancer patients may require greater energy support before, during and after treatment, as they are characterized by a number of metabolic changes (121). Proteins, lipids and especially carbohydrates are the nutrients that are required the most in cancer hypermetabolism, since they provide direct energy (122). Carbohydrates are one of the most necessary dietary components for humans, as they are the biomolecules that provide the essential glucose for energy supply (123).

In the past, although not so long ago, the idea of dogs and cats consuming carbohydrates was widely contested, with carbohydrates being seen as “villains” in the diet of these animals (13). Because the ancestors of these species did not have a diet rich in carbohydrates, it was believed that current consumption was responsible for the development of various comorbidities, including metabolic syndrome. However, the evolution of these species ensured the ability to metabolize and assimilate carbohydrates, incorporating them into their diet (124) and, until now, there are no scientific evidences proving that metabolic syndrome occurs and it is caused by carbohydrates in dogs and cats.

Glucose is the most common form of carbohydrate in nature and is an essential source of energy for various tissues. It can be obtained by diet, the endogenous reserve known as glycogen or synthesized via gluconeogenesis (125). This essential role of glucose becomes even more critical in dogs and cats diagnosed with cancer, particularly those that suffers from lack of appetite and paraneoplastic cachexia. In this case, an adequate nutritional intake that meets the requirements of an organism with altered metabolism becomes even more essential (126).

As previously mentioned, cancer cachexia is a syndrome that alters the patient’s functional status and is characterized by loss of body fat, muscle mass, appetite and systemic inflammation. Cachexia can be explained by the presence of a negative energy balance, which is the result of low food intake due to the side effects of chemotherapy treatments, as well as altered orexigenic neuropeptide signaling, possible taste alterations, and increased caloric needs in tumor-bearing animals (11, 71). Cancer cells are characterized by a distinct metabolism compared to normal cells, including an increase in glucose metabolism and, consequently, an increase in the activities of glucose transporter proteins and enzymes involved in energy consumption (127).

In 1920, Otto H. Warburg elucidated a tumor effect, which would explain where cancer cells obtained the greatest supply of energy for cell differentiation processes (128). The so-called “Warburg effect” describes a metabolic reprogramming of cells via the anaerobic glycolysis pathway, which results in a greater supply of glucose even in the presence of oxygen (129). A hundred years ago, the author of this theory, characterized by the conversion of glucose into lactate, pointed out that this effect was the result of a mitochondrial “defect,” thus generating a universal metabolic alteration. However, several others have emerged and it has been noted that in the majority of cancers, there are no disturbances at the mitochondrial level (130).

**Figure 1:**
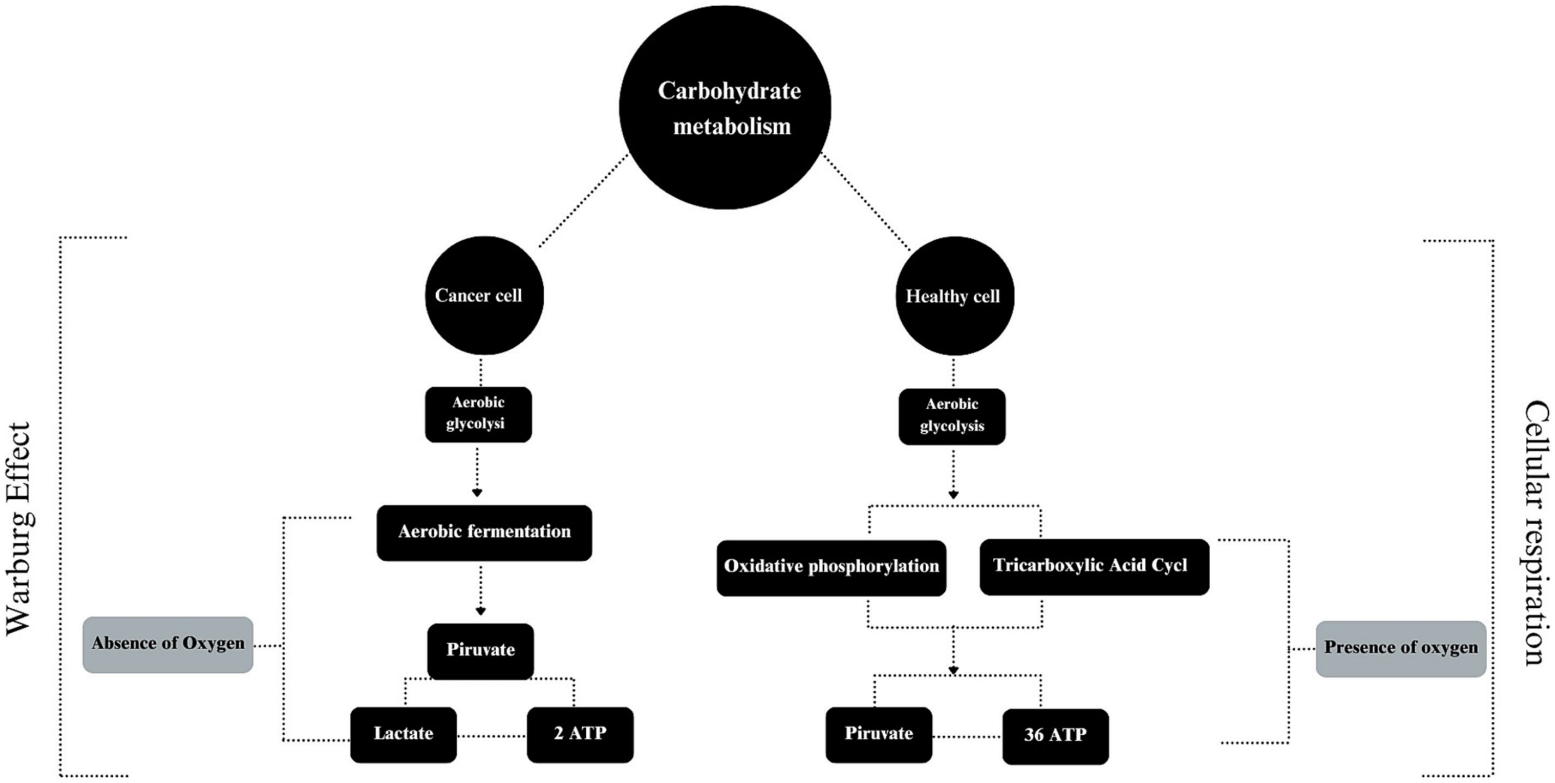
The use of carbohydrate by cancer and healthy cells.

In order to better understand the theme, it is essential to understand the characteristics of a cancer cell, which are: (a) stimulation of its own growth; (b) resistance to growth inhibitory mechanisms; (c) resistance to cell death itself; (d) stimulation of neoangiogenesis; (e) presence of abnormal metabolic pathways; (f) evasion of the immune system and; (g) high capacity to invade other tissues and organs (131). To carry out all these actions, a rapid energy supply pathway is required. Cancer cells therefore replace oxidative respiration with the fermentation of “sugar” (132), a mechanism already mentioned by Vail et al. (133) in a dog affected by lymphoma. In contrast, normal and healthy cells metabolize glucose via glycolysis into pyruvate, which is then sent to the tricarboxylic acid cycle (TAC) to generate a large amount of ATP molecules via oxidative phosphorylation (134). In cancer cells, anaerobic fermentation predominates despite its lower efficiency, producing only 2 ATP compared to 36 ATP molecules generated through oxidative phosphorylation. This rapid but less efficient process helps meet the high demands of rapidly dividing cancer cells (135) (Figure 1).

For this pathway to work effectively, it is essential that the uptake of glucose is high due to low performance of this ATP producing mechanism (136). To compensate, overexpression of glucose transporters (GLUT 1–4) occurs (137) along with activation of the pentose pathway and the development of insulin resistance (138). This mobilization is beneficial for the real purpose behind cancer’s preference for aerobic glycolysis: the production of metabolic intermediates for the synthesis of biomass, i.e., macromolecules essential for cell growth (139).

Understanding the mechanism of the Warburg effect is important to assimilate the “why” of the idea that: “nourishing the cancer patient means nourishing the growth of the tumor” (140). The metabolic alterations of carbohydrates against cancer cells are so significant that instead of supplying the host, the supply is diverted to the cancer, making it a kind of “parasite” (141). Through the Cori cycle, the lactate produced in excess by aerobic glycolysis is converted into glucose and, as a result, there is a gain of energy by the cancer and a net loss of energy by the host (142).

Therefore, with the aim of not providing the energy needed for tumor proliferation, various dietary modifications have been studied, mainly in relation to carbohydrate content intake. Given that cell proliferation takes place in an energy-rich environment, offering low-carbohydrate diets has emerged as an advantageous option for reducing the available “fuel” (143). In addition, the type of carbohydrate has also been discussed in terms of its impact on stimulating tumor proliferation (143).

High glycemic index carbohydrates, such as simple sugars, are not the most recommended options for cancer patients, as they trigger hyperglycemia, oxidative stress, glucose intolerance and stimulate an increase in insulin levels (144) and it is consistently reported in human literature, but not in veterinary literature. This increase in insulin promotes the activity of IGF-1, which also promotes cancer cell proliferation by inhibiting apoptosis (145). However, the consumption of complex carbohydrates is encouraged, as they have a lower glycemic index and regulate the activity of insulin-like factor binding protein 3 (IGFPB-3), which blocks the action of IGF-1, thus lowering tumor growth signaling (146).

With the discovery of the influence of carbohydrates, ketogenic diets have been attracting attention when it comes to the nutrition of cancer patients in human medicine (147). The diet itself is characterized by being high in fat and low in carbohydrates, creating an unfavorable environment for cancer cells and favorable for the anti-tumor effects of chemotherapy and radiotherapy (73). This effect was observed by Yang et al. (148), who identified the perfect combination of diet with chemotherapy, resulting in the suppression of pancreatic tumor growth. However, it has been observed that the effect of the diet may be specific according to the type of cancer and even so, the antineoplastic mechanisms are still poorly understood (149).

When it comes to dogs and cats, which are species that do not require carbohydrates and prefer proteins and lipids in their diet (150), removing carbohydrates will not lead to ketosis (as expected by ketogenic diets). As in humans, the high supply of fat, which is a source of fatty acids but has low amounts of other nutrients, will cause nutritional deficiencies if given in the amounts that are suggested in literature to promote ketosis, as reported by Vendramini et al. (15).

However, the idea of a low-carbohydrate diet for cancer-stricken dogs and cats is encouraged by some authors, with levels of 25% carbohydrates per dry matter (151). However, since the Warburg effect has not been confirmed in dogs and cats, the authors of this review suggest that reducing carbohydrates might be beneficial, as it could increase energy derived from fat and enhance the supply of protein, which is advantageous for patients with cachexia. Therefore, although the recommendations are the same, the underlying purpose differs.

Nonetheless, the total absence of carbohydrates in the dietary management of dogs and cats affected by cancer may not be encouraged, especially given the limited evidence linking carbohydrate intake to cancer recurrence (143). In one of the few studies evaluating a low-carbohydrate diet in dogs affected by lymphoma, no significant efficacy was observed in terms of cancer remission or increased survival time (152, 153). Some authors believe that the absence of these nutrients can compromise the search for other sources of energy, increasing the risk of loss of muscle mass and weight loss when the patient is still in negative energy balance. Thus, providing carbohydrates according to their glycemic index is the most appropriate way to determine the use of these biomolecules, and thus redirect their greater use toward the host and not the parasite.

### 3.2 Proteins and amino acids

The first step to understand the protein intake and specific amino acids supplementation for cancer patients, as well as to slow cancer-related cachexia, is early identification of cachexia-sarcopenia-malnutrition syndrome in clinical practice (154). As discussed previously, the overlapping mechanisms of lean body mass in cancer patients appear to be correlated with primarily inflammatory cytokines altering protein turnover, leading to both decreased synthesis and increased degradation (61). Considering nutritional support, the anabolic resistance demonstrated by patients could be partly reduced or prevented by increasing protein intake or supplementing specific amino acids (155). This section discusses proteins and amino acids with potential benefits for tissue repair, immune function and maintenance of lean mass for canine and feline with cancer.

Conventional nutrition is inefficient in minimizing cancer’s harmful degradation processes in the organism (156). Specific dietary factors support the prevention and reversal of cachexia and sarcopenia in humans and companion animals (157). Certain amino acids can serve as substrates for protein synthesis and help modulate the protein turnover and gluconeogenesis (62, 157). Further, studies demonstrated greater loss of lean body mass in dogs and cats that had lower protein intake (158, 159). Wakshlag et al. (158) reported loss of muscle mass and downregulation of p31 subunit, a regulatory protein in the 26S proteasome complex, in adult canines fed low protein diet over a 10-week period (158). The anabolic resistance could be overcome by increasing the amount of protein intake; however, “adequate” protein levels for dogs and cats also remain controversial.

In a randomized, blocked, controlled clinical trial by Laflamme and Hannah (159), 24 adult neutered male domestic shorthair cats were grouped by age, weight, and nine-point body condition score, and separated into three dietary treatment groups. The extruded dry diets were formulated to be as similar as possible, except for their protein and amino acid content. The diets contained 20, 26, and 34% dietary crude protein on an as-fed basis (21.9, 28.2, and 36.6% on a dry matter basis, and 5.6, 7.3, and 9.5 g protein/100 kcal diet, respectively), with essential amino acids maintained in consistent proportions while minimizing the use of crystalline amino acids. Following a 1-month baseline period, during which all cats consumed a 34% protein diet, the animals were fed the designated protein diets for 2 months. After 90 days, the authors (159) observed a linear increase in weight loss as protein intake was reduced, despite no difference in caloric intake among the cats.

The results indicated differences in the cat protein requirements for maintaining nitrogen balance and lean mass. Approximately 1.5 g protein/kg of body weight (2.1 g/kg^0.75^) is required to maintain nitrogen balance, while 5.2 g protein/kg of body weight (7.8 g/kg^0.75^) is needed to maintain lean body mass (159). The greater need for protein intake to maintain muscle mass is also observed in dogs (159). These same principles, in view of the greater need for protein intake, can be attributed to canine and feline cancer patients, as they suffer from cachexia and sarcopenia syndromes. Thus, adequate dietary protein intake is crucial for ensuring the maintenance of skeletal muscle mass (160, 161).

Interventions with amino acids have been tested in cancer patients to optimize nutritional status and counteract muscle mass wasting (160). For humans, these interventions include supplementation with branched-chain amino acids such as leucine, isoleucine, and valine, beta-hydroxy-beta-methylbutyrate, carnitine, and creatine (162). It is important to note that the digestibility and absorption of dietary protein sources significantly influence protein synthesis. Rapidly absorbed proteins have shown a greater anabolic effect because they quickly reach a peak concentration in the body. In contrast, protein sources that do not reach this peak result in lower protein synthesis (163).

Leucine is the most important branched-chain amino acid for preserving lean body mass (164). In dogs, the absence of branched-chain amino acids transaminase in hepatocytes prevents the liver from degrading those amino acids, resulting in most of the diet-derived branched-chain amino acids being utilized primarily by skeletal muscle. Thus, the availability of these nutrients for metabolic utilization depends of uptake by extrahepatic tissues (165). Leucine is considered the AAs with the greatest anabolic capacity (166), which can assist in regulatory actions in protein turnover and reduce proteolysis in the maintenance of lean body mass (167). There are no studies comparing tissue-specific or whole-body metabolism of branched-chain amino acids in cats.

Lysine deficiency can be attributed to increased protein catabolism and supplementation could reduce this degradation process (168). For dogs, it was observed that diets rich in lysine resulted in a decrease in the 20S proteasome, suggesting a reduction in protein catabolism (168). Frantz et al. (169) conducted a randomized, blocked and controlled clinical trial with thirty-six healthy geriatric cats. The animals were blocked by age, gender and body fat percentage and separated into four dietary treatment groups: food E (Purina ONE Senior Protection with 2.04% lysine); food F (experimental geriatric food with 1.65% lysine); food G (experimental geriatric food with 2.75% lysine) and food H (experimental geriatric food with 2.67% lysine and lower L-carnitine) for 90 day feeding period. The dietary protein content ranged from 6.87 to 10.22 g/100 kcal, while the lysine-to-calorie ratio ranged from 2.71 to 6.30. The authors observed that increasing dietary lysine, independent of total protein, helped reduce loss of LBM in aging cats. Supplementation with proteinogenic amino acids and high-quality functional nutrients such as taurine, 4-hydroxyproline, creatine, and carnosine (170) can be beneficial for oncologic dogs and cats.

Another critical issue for oncologic patients is the immunologic deficiency caused by the disease. In this regard, dietary protein and amino acids are crucial for immune system function. Certain amino acids, such as arginine, act as immunomodulators through various mechanisms in the body (171). Through various mechanisms and pathways, amino acids are essential for the proper functioning of the immune system, contributing to the synthesis of antibodies, cytokines, and glutathione (a potent antioxidant formed by glycine, cysteine, and glutamate) (171). Some studies could demonstrate the potential immunological properties of arginine and taurine, through nitric oxide synthesis, in combating pathogens (172, 173).

In a randomized controlled clinical trial conducted by Rutherfurd-Markwick et al. (173), 43 adult domestic short-haired healthy cats, males and females, intact and neutered and weighing 2.2–5.9 kg were divided into five groups, according to experimental diets: control group remained on the low protein diet (22.7% crude protein dry matter basis); positive control was fed a moist commercial high protein diet (53.0% protein dry matter basis), group 3 was fed the low protein diet supplemented with salmon oil; group 4 was fed the low protein diet supplemented with arginine (1.9% dry matter) and; group 5 was fed the low protein diet supplemented with nucleotides (9.25% dry matter) for a 35 days period. The authors observed that arginine supplementation caused a significant enhancement of lymphocyte proliferative responses to the T-cell mitogen phytohemagglutinin after 35 days and led to significant increases in blood leucocyte phagocytic activity after both 14 and 35 days (173).

In a randomized crossover study by Paßlack et al. (172), ten adults (7.37 ± 2.00 years old), healthy, females and males, intact and neutered cats were fed a three high-protein basal diet: basal diet (control); basal diet with arginine supplementation (50, 75, 100% compared to the arginine provision by the basal diet); and basal diet with ornithine supplementation (100, 150, 200% compared to the arginine provision by the basal diet). Blood sample collections were performed at the end of each 11-day treatment period to evaluate the possible effects of high-protein intake and arginine and ornithine supplementation on cat immune cells. The authors observed a quadratic effect for the dietary supplementation of arginine and ornithine in mitogen-stimulated proliferative activity of blood leukocytes, as well as a decrease in the percentage of phagocytic granulocytes. This effect was significant with increasing concentrations of arginine and ornithine (169). Adequate nutrition with amino acids such as lysine, cysteine, methionine, tryptophan, glycine, and proline may be crucial for enhancing both the innate and acquired immune systems, thereby reducing the risk of infection in animals (172).

**Figure 2:**
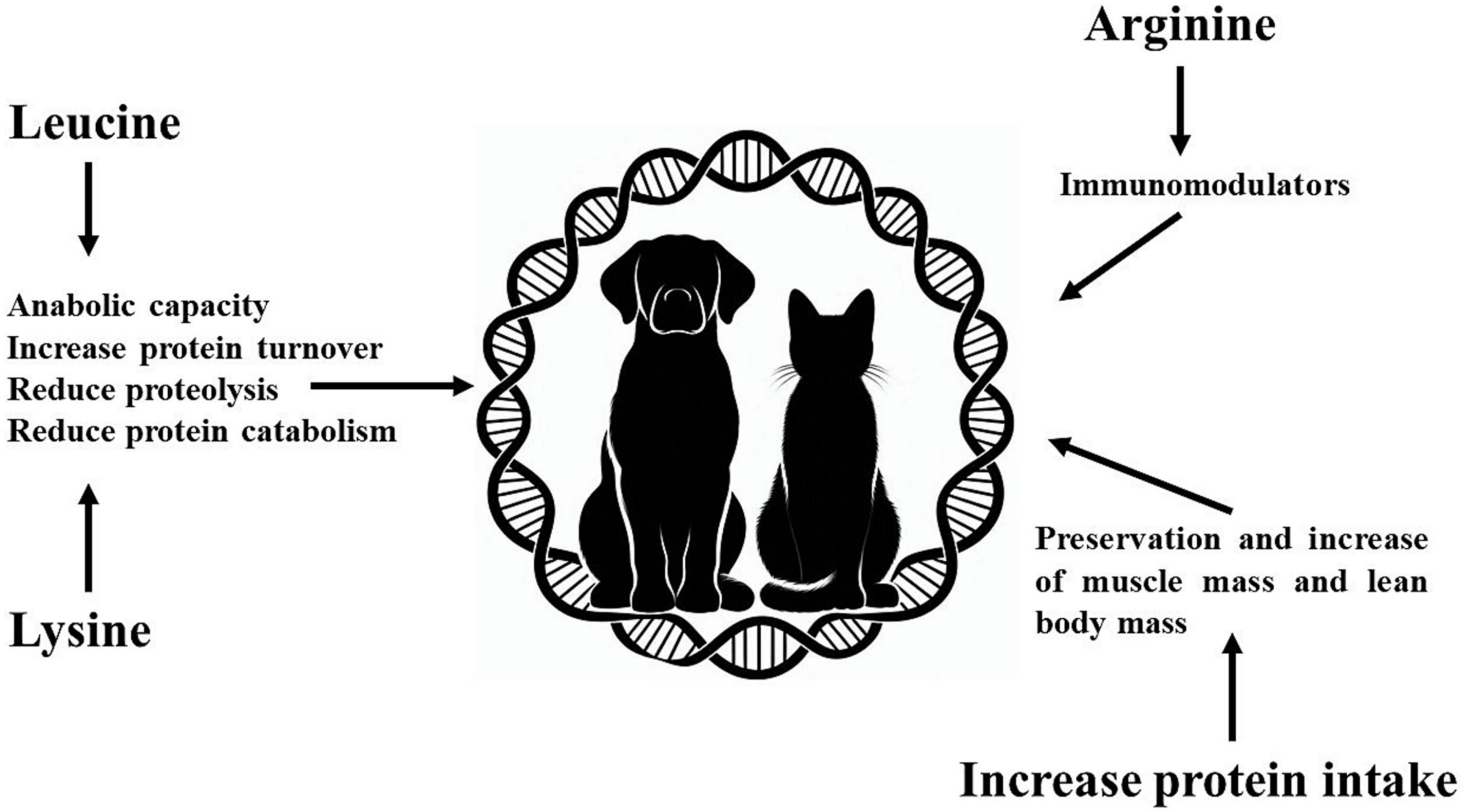
Effects of increasing protein intake and amino acids (arginine, leucine and lysine) in dogs and cats with cancer.

Dogs and cats with cancer often experience severe loss muscle mass due to cachexia or sarcopenia. Their diets should include adequate levels of high-quality and highly digestible protein (173), which may need to exceed National Research Council (174) and The European Pet Food Industry Federation (175) recommendations for dogs and cats. This is essential to ensure muscle protein synthesis and reduce muscle loss, with a particular emphasis on leucine and lysine supplementation. For human cancer patients, the recommended protein intake is 1.2 to 1.5 g/kg/day (176), 50% to 87.5% more than the recommendation for healthy individuals. For dogs and cats, the recommendation for cancer patients is protein intake of 1.0–1.2 g/kg/day evenly distributed during the day (61). Moreover, dogs and cats with cachexia or sarcopenia may require an increase in protein intake of 1.5–2.0-times the maintenance intake level to help mitigate the loss of muscle mass and lean body mass (61). The authors of this review also believe that arginine supplementation can improve the immune system capacity of these patients and can be an alternative to leucine and lysine supplementation (Figure 2).

### 3.3 Fat and essential fatty acids

Fatty acids are indispensable nutrients for dogs and cats, once they participate in several metabolic pathways and serve as an important source of energy and calorie storage (17). Beyond, the fatty acids are a part of the body’s inflammatory response, playing an important role in the development, morbidity and mortality of various diseases. These nutrients are key components of cell membranes and also function as signaling molecules in inflammatory processes (177). Based on these roles, the lipid profile of healthy dogs and cats may differ from that observed in oncologic patients (178).

Fats are more calorie-dense than carbohydrates and proteins, allowing the energy requirement to be met with a lower volume of food intake (179). In addition, they are considerably more palatable, which is especially interesting for cats that have a more selective appetite than dogs (180). The use of diets with higher fat and protein content facilitates spontaneous consumption by the animal (179) and improves the prognosis, since voluntary feeding is the preferred form of nutrition before opting for assisted enteral feeding. To this end, it is recommended that 50 to 60% of the total calories in the food come from fat (181), or, on a dry matter basis, that fat represents 25 to 40% (151).

The increase in energy density in the diet of cancer patients is interesting since these animals are in a state of hypermetabolism, which is characterized by a state of greater energy and oxygen expenditure (85). Oncogenic events, i.e., metabolic changes in the tumor environment, are responsible for significant changes in the way cells produce energy (182). Because the demand for oxygen is much higher than the supply, these microenvironments tend to exhibit hypoxia and nutrient scarcity. Therefore, cancer cells adapt to this environment by altering glucose metabolization, with high rates of glucose uptake and glycolysis, as well as changes in glutamine synthesis, which are essential factors in the growth and metastasis of tumors (183).

**Figure 3:**
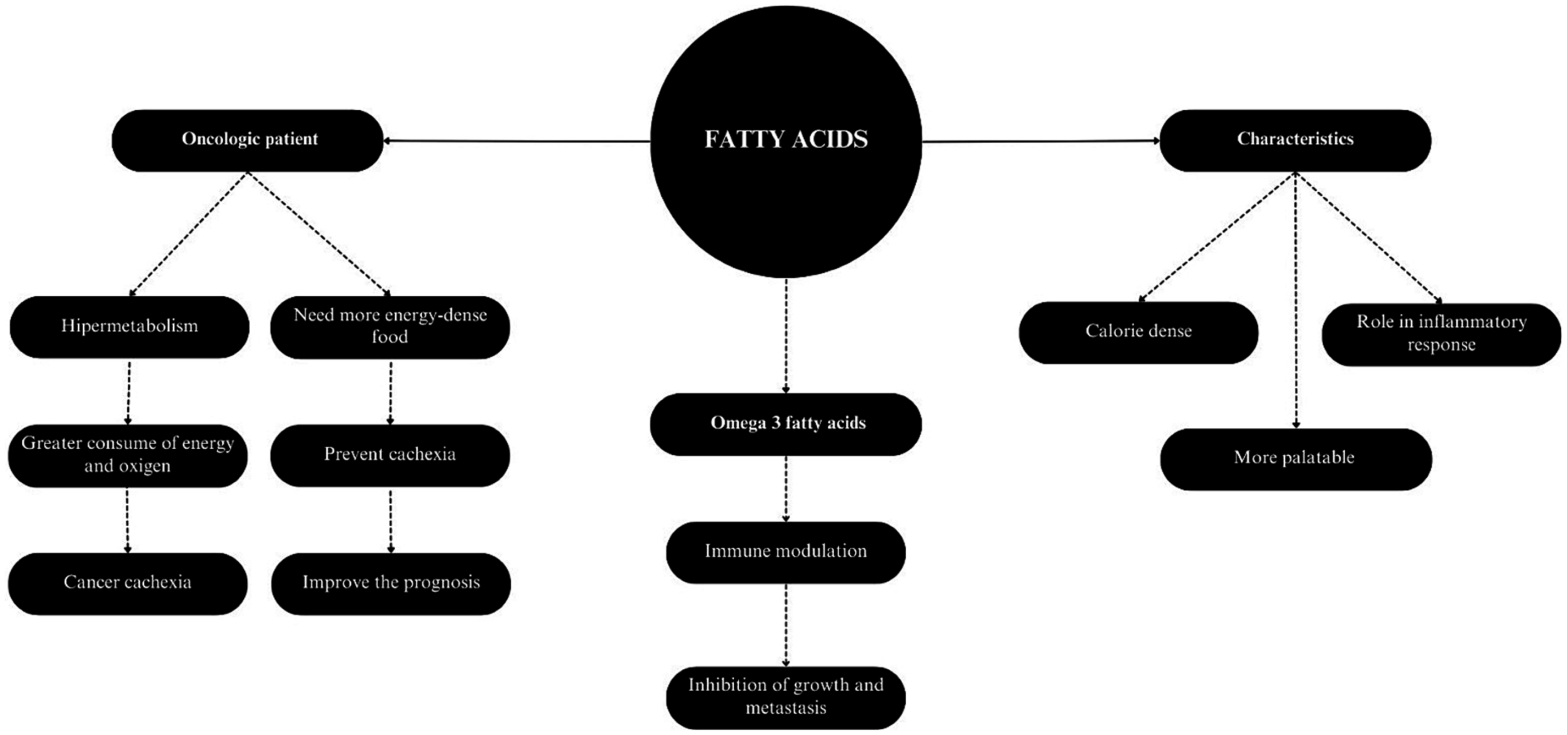
Characteristics and applicability of fatty acids in cancer patients.

In humans, fatty acids have an important role in the metabolism of individuals with cancer (Figure 3). Supplementation with specific fatty acids, such as omega-3 fatty acids, has been employed as an adjuvant therapy in the treatment of neoplastic diseases (184). These fatty acids are considered beneficial due to their function in immune modulation, since they participate in inflammatory reaction cascades similarly to omega-6, but producing less inflammatory and harmful mediators. Their incorporation may attenuate inflammation modulated by linoleic acid, which when chronic can increase the risk of cancer development in several organs (177). Omega-3 fatty acids influence the arachidonic acid cascade by inhibiting the formation of final metabolites such as prostaglandins, thromboxanes, and leukotrienes. Additionally, these fatty acids serve as precursors to anti-inflammatory mediators and promote the resolution of lesions (185, 186).

There are studies that claim that these fatty acids are related to the inhibition of tumor growth and metastasis. Olgivie et al. (84) suggested in his double-blind randomized study with 32 dogs diagnosed with lymphoma and undergoing chemotherapy, that the experimental diet containing menhaden fish oil, with an omega-3 to omega-6 ratio of 0.3:1 in dry matter, and supplemented with arginine, increased the survival and disease-free lifespan of the animals, compared to the placebo group that was fed an identical diet, but supplemented with soybean oil. The ratio between omega-3 and omega-6 recommended by Brunetto and Carciofi (187) ranges from 1:1 to 0.5:1 in dry matter for dogs and cats with cancer, which is confirmed by Saker and Selting (151), who also suggest providing levels of omega-3 fatty acids greater than 5% in dry matter.

In a study carried out on rats fed different unsaturated fatty acids of the omega-3 family, eicosapentaenoic acid (EPA) and docosahexaenoic acid (DHA) were shown to be related to a lower number and size of tumors in several organs, affecting carcinogenesis but without generating lesions in the liver, kidneys and bladder (187). The reduction in the lower incidence of these tumors was related to the lower levels of arachidonic acid in these organs, which is a substrate for inflammatory pathways.

Also, regarding polyunsaturated fatty acids of the omega-3 family, it is stated that dietary EPA and DHA can have protective action against several types of cancer, in addition to having an anti-cachexia effect (188). This effect is observed by several mechanisms: activation of apoptosis, suppression of cancer cell proliferation, and modifications in angiogenesis. These characteristics are interesting in the advancement of tools for the prevention and treatment of the most diverse types of cancer (189, 190).

Literature evidence supports the use of fatty acids as adjuvant chemotherapeutic agents, exhibiting different effects depending on the length of the aliphatic chain (191). *In vitro* studies have reported apoptosis-inducing activity across various cancer types; however, the optimal aliphatic chain length for maximum antineoplastic efficacy remains unclear. Short-chain fatty acids have been observed to induce apoptosis of cancer cells in *in vitro* studies (192). Alternatively, medium-chain fatty acids showed cytotoxic and antimicrobial properties in both *in vitro* and *in vivo* studies, but there is still little evidence whether this type of fatty acid has antineoplastic potential.

Finally, long-chain fatty acids such as linoleic acid also have antiproliferative properties in *in vitro* studies. There is concern that increased consumption of dietary linoleic acid may raise cancer risk, but human studies show no link between higher consumption of linoleic acid and increased cancer risk (193). The study regarding the use of these fatty acids as an alternative in the treatment of cancer is still in its initial phase, and further studies are needed to clarify the real antineoplastic effects of these molecules (191).

### 3.4 Fiber and digestive health

The role of fiber intake has become more studied in recent years. Adequate fiber consumption improves serum lipid levels, reduces blood pressure levels, enhances glucose control in patients with diabetes mellitus, aids in body weight loss programs, and supports the improvement of the immune system (194–197). In simplified terms, fibers are classified as either soluble, viscous, or easily fermentable in the colon or as insoluble fibers, which increase stool bulk but have limited fermentation in the colon (198). The positive effects of dietary fiber are partly related to the fact that some of its components are fermented in the large intestine. This fermentation impacts intestinal transit speed, colon pH, and the production of byproducts with important physiological functions (199).

Dietary fiber can help normalize colonic motility and transit time, support the normal growth of the gastrointestinal microbiota, and provide fuel for colonocytes (200). Soluble fibers can be added to a normal diet to improve stool consistency due to their high water-retention capacity, forming gels in water. They can also influence colonic function by increasing the weight and absorptive area of the colon (201). The microbiota also ferments soluble fibers and produces short-chain fatty acids, which promote the regeneration of colonocytes and may improve recovery from diarrhea (202). Insoluble fibers do not form gels, and their fermentation is limited. However, they has the ability to modulate gastrointestinal motility and is also associated with mucosal hypertrophy in the colon, which is accompanied by an increase in colonic weight, suggesting a possible enhancement in absorptive capacity (203). Lignin, cellulose, and some hemicelluloses are insoluble fibers (204).

Since dietary fibers are not absorbed, their effects would be associated directly in the intestine and indirectly in the overall system by the action of their fermentation products. For this reason, fibers are well investigated in diseases of the gastrointestinal tract, such as colon cancer and colorectal cancer in humans (205). Colon rectal cancer is considered one of the cancers most directly associated with dietary habits, especially high consumption of protein and low intake of fibers (206). There is correlation between low fiber intake and the risk of developing intestinal cancer in humans.

This association involves multiple pathways, including the effects of fiber on bile acid metabolism, its vegetable sources that may contain antioxidants such as resveratrol and polyphenols, and its prebiotic effects that modulate gut microbiota. This modulation leads to the production of short-chain fatty acids, such as butyrate, acetate, and propionate, which are crucial for maintaining intestinal cells, adhesion molecules, and immunoregulation, particularly given the proximity of the gut-associated lymphoid tissue (205, 207). Furthermore, the increase in the production of short-chain fatty acids as a result of fermentation leads to a decrease in intracellular and colonic pH (208, 209). The more acidic environment helps protect the intestines, inhibits the proliferation of pathogenic organisms, and reduces the formation of toxic products that may correlate to carcinogenesis such as putrescins, produced by some bacteria using protein as a substrate.

In dogs and cats, there are no established minimum values for fiber intake, as fiber deficiency does not usually cause nutritional deficiencies, and there is therefore no evidence to associate cancer with low fiber intake in small animals. However, in humans with cancer, a minimal inclusion of both soluble and insoluble fibers is recommended for optimal intestinal health, with a suggested intake of 30–35 g/day for adults (174, 205). As in humans, dogs and cats with cancer require adequate gut health to optimize food digestibility. To achieve this, it is essential to provide a sufficient amount of fiber, which can be achieved with crude fiber levels in dry matter above 2.5% (187). Although these values are relatively low, they can be effective when combined with high-quality fibers that promote intestinal fermentation and the production of volatile fatty acids, which help to improve lipid and glucose metabolism.

Although the authors discuss the impact of high fiber on digestibility, they do not specify a maximum threshold. Maintenance diets typically contain less than 5% fiber, while a recent study classified a diet with 7.8% fiber as high-fiber (210). Therefore, maintaining fiber levels within this range may be ideal.

Considering these effects and the others mentioned, more studies, especially experimental ones, are needed to more accurately evaluate the importance and quality of fibers in dogs and cats with cancer, making them targets for future research.

### 3.5 Crucial vitamins and minerals

#### 3.5.1 Vitamin D

Vitamin D is an essential nutrient for cats and dogs (174, 175) and has a fundamental role in bone and calcium metabolism. Further, vitamin D has shown important effects in non-bone-related diseases, including cancer (211–213). The role of vitamin D in cancer has been largely studied in the past years (214, 215). In humans, vitamin D deficiency has been associated with the occurrence and progression of different types of cancer (216–219), and vitamin D supplementation has shown beneficial effects in the prevention and clinical outcome of cancer (220–223). Previous studies have demonstrated anti-inflammatory, immunomodulatory, and antitumoral effects of vitamin D in cancer metabolism in multiple pathways. These include inhibition of tumor growth and proliferation, induction of apoptosis in cancer cells, downregulation of the nuclear factor kappa b (NF-κB) pathway, cytokine regulation, upregulation of MAP kinase phosphatase 5 (MKP5), and inhibition of cyclooxygenase 2 (COX-2) (224–227).

A systematic review and meta-analysis by Zhang et al. (215) assessed supplementation of vitamin D and its association with all-cause mortality in 50 clinical trials involving 74,655 human adults. The study found no significant association between vitamin D supplementation and total mortality; however, it was linked to a 16% reduction in cancer mortality. Kuznia et al. (226) performed a systematic review and meta-analysis of the effects of vitamin D3 supplementation on cancer mortality in 14 placebo-controlled trials. Although the overall analysis found that vitamin D3 supplementation did not significantly reduce cancer mortality, a restricted analysis focusing on studies with daily dosing regimens revealed different results. Daily supplementation was associated with a 13% reduction in cancer mortality and an 11% increase in cancer-specific survival.

Weidner et al. (227) analyzed plasma 25-hydroxyvitamin D (25(OH)D) concentrations in dogs with osteosarcoma, lymphoma, and mast cell tumor, and found that cancer type was correlated to 25(OH)D concentrations. Cancer patients with higher plasma ionized calcium concentrations presented lower 25(OH)D concentrations. Additionally, plasma concentrations of 24,25-dihydroxyvitamin D (24,25(OH)2D), which was influenced by dietary intake of vitamin D, also had significant impact on plasma 25(OH)D.

Sadeghian et al. (228) evaluated serum 25(OH)D and parathyroid hormone levels in dogs with transmissible venereal tumor, and found significantly lower 25(OH)D and higher parathyroid hormone concentrations compared to healthy dogs. Selting et al. (229) compared serum 25(OH)D concentrations between healthy dogs and those with acute hemoabdomen, the latter with various types of cancer, primarily splenic hemangiosarcoma. The study concluded that the relative risk for splenic hemangiosarcoma decreased as 25(OH)D concentration increased and found a potential protective effect of 25(OH)D in concentrations over 100 ng mL^−1^. Furthermore, the authors suggested a target concentration between 110–120 ng mL^−1^ for 25(OH)D in dogs.

Wakshlag et al. (230) investigated the association between serum 25-hydroxyvitamin D_3_ (25(OH)D_3_) concentrations and cutaneous mast cell tumors in Labrador retrievers. The authors found significantly lower serum 25(OH)D_3_ in the mast cell tumors group when compared to healthy dogs, and no difference in vitamin D intake between the groups. It is important to highlight that clinical studies evaluating the benefits and safety of vitamin D supplementation for cats and dogs with cancer are yet to be performed (213). However, there are some studies investigating the possible antineoplastic effect of vitamin D in dogs, such as the study conducted by Malone et al. (231), who observed, through the administration of 2.25 μg of calcitriol/kg per week, complete remission, in addition to three partial remissions with the dose of 1.5 μg of calcitriol/kg per week, in dogs diagnosed with mastocytoma. However, despite this result, most of the animals in the study showed signs of toxicity at the dose, which led to the need to discontinue the study. Therefore, new studies are needed to investigate more appropriate doses of this vitamin.

Currently, there is no specific dose recommendation for vitamin D supplementation for cancer in small animals or any other disease. Nevertheless, it is crucial to distinguish between dietary requirements and actual intake. While this can be challenging in human studies, it is more manageable in veterinary studies with dogs and cats, which typically consume commercial diets with declared compositions and more controllable or estimable feeding amounts (232). Defining an appropriate dose for supplementation is essential for all types of supplements, not just vitamin D. Consequently, dogs and cats, as target species and animal models, may benefit from these findings.

#### 3.5.2 Vitamin C

Vitamin C is a water-soluble vitamin that acts as an antioxidant. It is considered an essential nutrient for humans, but non-essential for healthy dogs and cats, as they are able to synthesize it in the liver (233) and thus, low dietary intake does not produce deleterious effects in these species (22).

In human cancer patients, the administration of high doses of vitamin C has been suggested as potentially beneficial, mainly by intravenous administration; however, this approach is controversial and has yielded mixed results (234). In past studies, vitamin C has shown anticancer effects by killing or inhibiting the growth of tumoral cells both *in vitro* and *in vivo* (235–238). A study by Shin et al. (239) found that ascorbate was able to reduce the viability of canine melanoma cells *in vitro.*

The actual significance of vitamin C supplementation for human cancer patients is yet to be determined (234, 240–242). Dogs undergoing chemotherapy and fed a diet containing 280 ppm of vitamin C per dry matter showed weight gain and good acceptance of the food. However, these results were more related to the other nutrients in the diet than specifically to the vitamin C present in the composition (29). More studies regarding cats and dogs would be necessary to understand whether any beneficial effects of supplementation for cancer would exist in these species (22).

#### 3.5.3 Zinc

Considered essential for cats and dogs (174), zinc is a trace element that participates in multiple enzymatic reactions and processes such as cell replication and protein metabolism (243, 244). In small animals, zinc deficiency has been associated with skin lesions and growth impairment (245–247). Besides, zinc has been studied for its role in inflammation and immunity (248–250). In humans and animal models, the association between zinc deficiency and cancer has been reported (251–255). Moreover, zinc has shown anticancer effects by various pathways, including immune system modulation, inhibition of NF-κB, antioxidant effects, and apoptosis regulation (256–258).

Kazmierski et al. (259) evaluated serum concentrations of zinc, chromium and iron in dogs with lymphoma and osteosarcoma. The authors found decreased zinc concentrations in both osteosarcoma and lymphoma patients, when compared to healthy dogs. Chromium and iron concentrations were also lower in both cancer types. Brodzki et al. (260) compared antioxidant capacity and serum zinc concentration in dogs with malignant and non-malignant perianal tumors, before and after antihormonal treatment. Dogs with malignant tumors presented significantly lower zinc concentrations before treatment and higher concentration after treatment when compared to dogs with the control group. The researchers suggested that zinc concentrations could have clinical use, as low zinc concentrations were associated with malignant tumors, and increasing concentrations after treatment were associated with worse prognostic.

A study by Zhang et al. (261) evaluated post-diagnosis zinc supplementation for prostate cancer in men and found that lower doses (1–24 mg/day) were associated with a decreased risk of all-cause mortality and lethal prostate cancer. However, another study by Zhang et al. (262) found that supplementation in higher doses (>75 mg/kg) for more than 15 years was related to higher risk for lethal and aggressive prostate cancer. Thus, more studies are necessary to assess the need and potential risks and benefits of zinc supplementation in cancer patients.

Therefore, supplementation is currently not considered necessary for cancer patients if they are consuming a diet with balanced zinc levels, as recommended by the NRC (24, 174). For adult dogs, the recommended dose is 1.0 mg/kg body weight/day. For cats, the recommended doses are 1.2 mg/kg of body weight/day for adults.

#### 3.5.4 Other nutrients

Other vitamins and minerals have been studied for cancer patients, such as selenium and vitamin E. Selenium has shown potential anticancer effects such as selective cytotoxicity, antioxidant effects and inhibition of metastatic cell migration (263–267). Waters et al. (268) demonstrated that at a dose of 3 μg/kg/day or 6 μg/kg/day, selenium may have a protective effect on prostate cell DNA and may promote epithelial cell apoptosis, helping to prevent prostate cancer in aging dogs. However, conflicting results exist, with some studies reporting an association between selenium and increased cancer progression and risk (269–271).

Vitamin E, known for its established antioxidant roles, has been evaluated for cancer prevention. *In vivo* and animal studies have demonstrated its ability to induce autophagy and apoptosis in cancer cells, as well as to modulate the antitumor immune response (272–276). A retrospective study in humans by Yuan et al. (272) found that cancer patients supplemented with vitamin E presented increased survival rates and better response to treatment. In dogs with lymphoma, Winter et al. (277) found lower serum concentrations of alpha and gamma-tocopherol before treatment, and an increase in alpha-tocopherol after treatment. Karayannopoulou et al. (278) did not find significant differences in serum alpha-tocopherol in dogs with mammary gland tumors, but found lower concentrations in neoplastic tissue when compared to healthy mammary tissue. Finotello et al. (279) also found no difference in serum alpha-tocopherol concentrations for dogs with mammary gland tumors.

However, the antioxidant effects of vitamin E in diets containing 500, 1,000 or 1,500 IU/kg, associated with vitamin C (100 ppm) and *β*-carotene, have already been demonstrated in dogs and cats (280), although it has not yet been evaluated in animals with cancer. Even so, these results indicate the potential benefits of using this vitamin, associated with other antioxidants, in cancer patients.

There has been ongoing discussion among veterinary oncologists about vitamin B12, particularly concerning its supply, as some metabolic pathways for cancer growth are favored by the presence of B12 (281). Some literature has pointed out that high concentrations of B12 have been found in patients with hepatic cancer, and this correlation has been contested and refuted. Cyanocobalamin is the only vitamin in the B complex that is stored in the liver; therefore, liver diseases, especially cancer, may lead to elevated blood concentrations of B12 if the storage capacity is compromised (282) According to a systematic review by Obeid (282), there are no evidence that links plasma B12 to cancer.

The idea of withholding certain nutrients from cancer patients has been discussed in other fields of nutrition. This concept emerged from discussions about the Warburg effect and subsequent discoveries. Walburg discovered that tumor cells have more antioxidant capacity than normal cells and use a faster pathway to produce ATP, anaerobiosis, as mentioned before. After discovering that the induction of IGF-1 production activates a pathway related to chemotherapy resistance (PIK3/AKT) and transportation to lactate from inside the cell outside, favoring tumor growth, researches have tried to develop strategies to overcome this issue, by simple starvation (72 h fasting prior to chemotherapy) or through starvation mimetics (nutraceuticals, for example), which are the compound that blocks this pathway or others related to, as the SGLT-2, reducing glucose uptake by cancer cells (283, 284).

As serum B12 are evolved in glycolysis, and B12 deficiency leads to lower production of IGF-1, inducing it could have passed by the understanding that it could be useful (285). Inducing deficiencies in cancer patients, whether related to energy or nutrients, have quickly counterbalanced this recommendation with a simple justification: it is not wise to induce starvation in a sick patient. The immune system and other systems require nutrients and energy to keep the patient alive. Therefore, based on the reasons for withholding B12 supply, the authors of this work also support recent conclusions (282) that suggest ending this recommendation. While this may not be a concern for patients on conventional complete dry and extruded diets, it could be an issue for patients following unconventional diets—common in cancer care—that require supplementation, including vitamin B12 especially in a different species.

Thus, for dogs and cats with cancer, supplementation may be necessary when there is no optimal supply of this vitamin in the diet, with an intake of 4.4 μg per 1,000 Kcal of metabolizable energy being recommended for adult cats and 9.6 μg per 1,000 Kcal of metabolizable energy for adult dogs (175).

The findings discussed here suggest that vitamins and minerals could play a significant role in cancer therapy. While promising, the therapeutic use of nutrients like selenium and vitamin E requires more research to establish their efficacy and safe application (286). As the study of these nutrients evolves, new insights may emerge, expanding their potential as complementary options in the cancer treatment of dogs and cats. Below, Table 1 is presented with a summary of the nutritional needs discussed in this work.

**Table 1:** Key nutritional recommendations for dogs and cats with cancer.

Nutrients	Authors	Recommendation
Carbohydrates	Saker e Selting (151), Clemente et al. (146)	Inclusion of complex carbohydrates or a total of 25% carbohydrates in DM*
Proteins and amino acids	Deutz et al. (176), Paßlack et al. (172)	Dogs and cats with cancer require higher protein intake (1.0 to 1.2 g/kg/day) and supplementation with essential amino acids
Fat and fatty acids	Saker and Selting (151), Case et al. (181), Brunetto and Carciofi (187)	50 to 60% of the total calories of the food or 25 to 40% fat in DM > 5% omega-3 fatty acids in DM omega-6: omega-3 between 1:1 and 0.5:1 in DM
Fiber	Rossi et al. (210), Brunetto and Carciofi (187)	> 2.5% and < 7.8% crude fiber in DM
Vitamins	Malone et al. (231), Jewell et al. (280), FEDIAF (175)	*Vitamin D: 1.5 to 2.25 μg calcitriol/kg per week in dogs with mastocytoma Vitamin C: Between 100–280 ppm Vitamin E: 500, 1,000 or 1,500 IU/kg Vitamin B12: adult cats: 4.4 μg per 1,000 Kcal of metabolizable energy. adult dogs: 9.6 μg per 1,000 Kcal of metabolizable energy
Minerals	Waters et al. (268), NRC (174), Rataan et al. (266)	Zinc: Adult dogs: 1.0 mg/kg/day. Adult cats: 1.2 mg/kg/day Selenium: 3 μg/kg or 6 μg/kg in dogs

DM, Dry matter; *Vitamin D: dose toxicity warning.
