# Correction: Connection between nutrition and oncology in dogs and cats: perspectives, evidence, and implications—a comprehensive review

**DOI:** 10.3389/fvets.2026.1919852

**Published:** 2026-07-22

**Authors:** Andressa R. Amaral, Gabriela L. F. Finardi, Pedro H. Marchi, Natália M. C. de Oliveira, Leonardo A. Príncipe, Natacha Teixeira, Maria C. F. Pappalardo, Laís O. C. Lima, Juliana V. Cirillo, Júlio Cesar de C. Balieiro, Thiago H. A. Vendramini

**Affiliations:** 1 Veterinary Nutrology Service, Veterinary Teaching Hospital of the School of Veterinary Medicine and Animal Science, University of São Paulo, São Paulo, Brazil; 2 Pet Nutrology Research Center, Department of Animal Nutrition and Production of the School of Veterinary Medicine and Animal Science, University of São Paulo, Pirassununga, Brazil

**Keywords:** appetite, cachexia, cancer, canine, feline, food, nutrology

In the published article, there was a mistake in Section 3.2 *Proteins and amino acids*, Paragraph 11. The citations for the protein intake recommendations were incorrectly assigned. In addition, the final sentence of the paragraph lacked a reference and has been revised to more accurately reflect the recommendations described in the cited veterinary source.

A correction has been made to the Section 3.2 *Proteins and amino acids*, Paragraph 11:

“For human cancer patients, the recommended protein intake is 1.2 to 1.5 g/kg/day (176), 50% to 87.5% more than the recommendation for healthy individuals. For dogs and cats, the recommendation for cancer patients is protein intake of 1.0–1.2 g/kg/day evenly distributed during the day (61). Moreover, dogs and cats with cachexia or sarcopenia may require an increase in protein intake of 1.5–2.0-times the maintenance intake level to help mitigate the loss of muscle mass and lean body mass (61).”

The original version of this article has been updated.

